# Mechanobiology of the relocation of proteins in advecting cells: in vitro experiments, multi-physics modeling, and simulations

**DOI:** 10.1007/s10237-023-01717-2

**Published:** 2023-04-17

**Authors:** M. Serpelloni, M. Arricca, C. Ravelli, E. Grillo, S. Mitola, A. Salvadori

**Affiliations:** 1 The Mechanobiology research center, UNIBS, 25123 Brescia, Italy; 2 Department of Mechanical and Industrial Engineering, Università degli Studi di Brescia, 25123 Brescia, Italy; 3 Department of Molecular and Translational Medicine, Università degli Studi di Brescia, 25123 Brescia, Italy

**Keywords:** Mechanobiology, Chemo-mechanical-transport, Finite strains, Non-equilibrium thermodynamics, High-performance computing, Finite elements

## Abstract

Cell motility—a cellular behavior of paramount relevance in embryonic development, immunological response, metastasis, or angiogenesis—demands a mechanical deformation of the cell membrane and influences the surface motion of molecules and their biochemical interactions. In this work, we develop a fully coupled multi-physics model able to capture and predict the protein flow on endothelial advecting plasma membranes. The model has been validated against co-designed in vitro experiments. The complete picture of the receptor dynamics has been understood, and limiting factors have been identified together with the laws that regulate receptor polarization. This computational approach might be insightful in the prediction of endothelial cell behavior in different tumoral environments, circumventing the time-consuming and expensive empirical characterization of each tumor.

## Introduction

Cancer growth is associated with an abnormal development of blood vessels, a process named tumor angiogenesis. Blood vessels deliver inside the tumor, in addition to oxygen and nutrients, the endothelial cell (EC) precursors such as the circulating endothelial cells (CEC) and the bone marrow derived-endothelial progenitor cells (EPCs). These cells differentiate into mature ECs supporting the tumor neovascularization (tumor vasculogenesis). The recruitment and mobilization of CEC and EPCs are driven by angiogenic growth factors and chemokines, such as vascular endothelial growth factor (VEGF) and gremlin (Goon et al. 2006; Raz et al. 2014; Tanaka et al. 2019; Wang et al. 2019).

After activation, EPCs adhere to the neoplastic tissue, polarize, and differentiate into mature ECs. Several membrane receptors (e.g., integrins, growth factor receptors, and cadherins), able to relocate on the cell membrane and to transduce extracellular signals inside the cell, collaborate in the EPCs maturation and new vessel formation. Among them, the VEGF receptors (VEGFRs) play a central role in EPCs migration and vasculogenesis (Ash and Overbeek 2000; di Somma et al. 2020). Here, we focus on the ability of *gremlin*, a VEGFR2 ligand, to induce the *relocation of the receptor on an advecting EC plasma membrane* (Ravelli et al. 2015). Gremlin is a soluble molecule secreted in the tumor microenvironment both by ECs and parenchymal cells (Sneddon et al. 2006). The heparin-binding domain of gremlin (Chiodelli et al. 2011) allows its accumulation in the extracellular matrix (ECM), where it stands as a long-lasting stimulus for ECs.

The rapid cellular remodeling and the complexity of the plasma membrane microdomains, typically smaller than the diffraction limits of optical microscopes, make it hard to outline the receptor dynamics (Huang et al. 2017). We describe herein the VEGFR2 dynamics during EC adhesion combining *in vitro* experiments with computational models. A fully coupled multi-physics theory has been developed to this purpose. Numerical simulations predict the concurrent protein flow and the advection of the plasma membrane in ECs.

The model is framed in the mechanics and thermodynamics of continua at finite strains (Gurtin et al. 2010), with chemical kinetics and transport equations defined on curved manifolds embedded in higher-dimensional spaces. Rigorous and nowadays classical thermodynamic strategies (energy and entropy balance, the choice of the Helmholtz free energy as thermodynamic potential, the Coleman–Noll procedure) guide the constitutive modeling.

Co-designed experiments and simulations concern cellular adhesion on a rigid $\mu$slide coated with ligands. Adhesion induces the transport of specific receptors from the apical to the basal part of the cell, generating attractive forces. In this work, we show that electrostatic interactions cannot be the mere responsible for ECs spreading, which shall be attributed to the protrusion of the leading edge, retraction, and contraction (Doyle et al. 2021).

Although several studies attempted at capturing the processes that drive cell motility (see Bonanno et al. 2023;  Serpelloni et al. 2021) and literature therein), a comprehensive model of the cytoskeletal machinery is not yet available in the literature. This manuscript does not attempt at proposing a new theory either. By adopting finite strain contact mechanics, we eventually made a digital twin of a complex cellular experiment, unveiling that a fast interaction due to chemical bonding at adhesion precedes a mechanically dominated regime, in which free receptors are engaged by their ligands during the mechanical evolution of the cell. Finally, once a *macroscopic* steady-state mechanical configuration has been achieved, transport of receptors on the membrane continues and favors complex localization at the edges of cell/ECM contact area (Damioli et al. 2017; Salvadori et al. 2018).

The present paper is designed as follows. Section 2 contains materials and methods for the experimental procedures, as well as modeling definitions. One of the main conclusions of this paper is presented in Sect.3: we show, experimentally and theoretically, that electrostatic interactions cannot be responsible for cell spreading of ECs, in view of the modest amount of energy involved in those interactions compared to the bulk energy of a cell. This claim guides the design of a digital twin for the relocation of proteins on lipid membranes. We study in Sect.4 the spreading of an EC as driven by the cytoskeletal machinery rather than electrostatic interactions. The developed multi-physics model aims at reproducing the relocation of VEGFR2 on the advecting membrane, with particular emphasis on chemo-transport processes during the cell spreading on a glass ${\mathrm{\mu }}$slide. The outcomes of the high-performance computing simulations allow validating the multi-physics model against a complex experimental setup, eventually showing remarkable predictive performances.

**Fig. 1:**
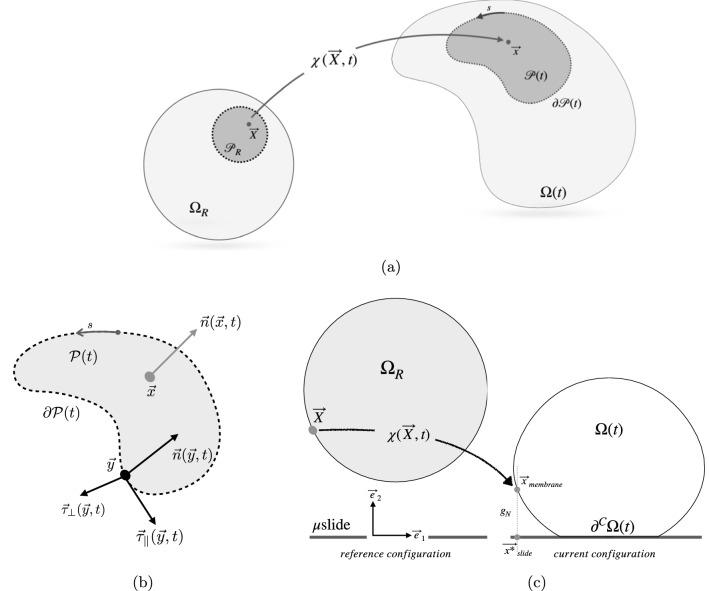
Notation. **a** The reference $\Omega _R$ and the deformed cell $\Omega (t)$. Note that $\vec {x} \in {{{\mathcal {P}}}(t)}$ implies $\vec {X} \in {{{\mathcal {P}}}_R}$. **b** Frenet frame $\{ \vec {\tau }_{\parallel }, \vec {\tau }_{\bot }, \vec {n} \}$ at point $\vec {y} \in \partial {{\mathcal {P}}}(t)$. *s* is the arc length measured on $\partial {{\mathcal {P}}}(t)$ (Serpelloni et al. 2022). **c** The notation for cell adhesion and spreading, drawn in 2D for the mere sake of readability

## Notation and multi-physics model definition

### Notation

The notation for scalars, vectors, and tensors is summarized in Table 1. Lowercase fonts denote $\vec {vector}$ and ${\varvec{tensor}}$ fields in the current configuration, whereas uppercase letters are used for referential $\vec {VECTOR}$ and ${\varvec{TENSOR}}$ fields. A different notation holds for scalar fields, consistently with (Gurtin et al. 2010), which are denoted with a subscript $_{R}$ in the reference configuration. The reason for this choice is that uppercase letters either denote parameters (as per the shear modulus *G*), or customary different scalar fields ( as per temperature *T* versus time *t*, or concentration *c* and the chemical species *C*).

**Table 1:** Notation adopted for tensor, vector, and scalar fields both for current and reference configuration

Variables and values	Reference configuration	Current configuration
2-nd order tensor	${\varvec{A}}$	${\varvec{a}}$
Vector	$\vec {A}$	$\vec {a}$
Scalar	$a_R$	*a*

### Definitions

Denote with $\Omega (t)$ an advecting cell and with $\partial \Omega (t)$ its surface. As depicted in Fig. 1a, a position $\vec {x} \in \Omega (t)$ is the image of a point $\vec {X}$ in a reference configuration $\Omega _R$ through a smooth function ${\chi }(\vec {X},t)$ termed *motion*. We will name *deformation* ${{\chi }}_t(\vec {X})$ the snapshot of a motion at a fixed time *t*. The deformation is assumed to be a one-to-one map and its *referential* gradient ${\varvec{F}}$ must have a strictly positive determinant $J = {\det } \left[ \, { {\varvec{F}} } \, \right] > 0$. Denote the binormal vector $\vec {n}$ — see Fig. 1b — as the image in ${{\mathcal {P}}}(t)$ of a vector $\vec {n}_R$ in the reference configuration ${{\mathcal {P}}}_R$, through the contravariant transformation

$$\begin{aligned} \vec {n} = {\varvec{F}} ^{-T} \, \vec {n}_R \;. \end{aligned}$$

The *projected referential gradient operator* of a scalar field $f({\vec {X}}, t)$ on ${{\mathcal {P}}}_R(t)$ is defined as follows

1a $$\begin{aligned} \textrm{Grad}_{ {{\mathcal {P}}}_R } \left[ \, { f } \, \right] = \textrm{Grad} \left[ \, { f } \, \right] - \; \frac{ \vec {n}_R \cdot \textrm{Grad} \left[ \, { f } \, \right] }{ | \vec {n}_R |^2 } \, \vec {n}_R, \end{aligned}$$

whereas the *projected referential divergence operator* of a vector field $\vec {v}({\vec {X}}, t)$ on ${{\mathcal {P}}}_R(t)$ is defined as

1b $$\begin{aligned} \textrm{Div}_{ {{\mathcal {P}}}_R } \left[ \, { \vec {v}_R} \, \right] = \textrm{Div} \left[ \, { \vec {v} } \, \right] - \; \frac{ \vec {n}_R \cdot { \textrm{Grad} \left[ \, {\vec {v}_R} \, \right] } \vec {n}_R }{ | \vec {n}_R |^2 }. \end{aligned}$$

Tractions (forces per unit area) on the boundary are the idealization of the mechanical interaction between a solid and its surrounding (Gurtin et al. 2010). They can be categorized in *surface and contact*. The amount of the former is given provided that the configuration is known. The value of the contact tractions, on the contrary, is arbitrary even if the configuration is known; it is part of the solution of a set of differential governing equations.

Contact and surface forces are required to model experimental tests on ECs that concern adhesion and spreading onto a glass-made $\mu$slide. Figure 1c depicts the basic notation for the problem. Particularly, the minimum distance $g_N$ between a point on the cell membrane and the glass-made $\mu$slide has been depicted, in this figure, through the frontal view of the 3D mechanism of cell adhesion and spreading. Since glass-made $\mu$slides of interest are flat, the normal vector at a generic point $\vec {x}_\mathrm{{slide}} \in \mu {\textrm{slide}}$ is $\vec {n}_\mathrm{{slide}} = \vec {e}_2$ and it remains unaltered in time. Accordingly, a single point $\vec {x^*}_\mathrm{{slide}} \in \mu {\textrm{slide}}$ is associated with a corresponding point $\vec {x}_\mathrm{{membrane}} \in \partial \Omega (t)$ via the minimum distance method, by projecting $\vec {x}_\mathrm{{membrane}}$ onto the $\mu$slide. The inequality constraint

2 $$\begin{aligned} {{\textit{g}_N}} = ( \vec {x}_\mathrm{{membrane}} - \vec {x^*}_\mathrm{{slide}} ) \cdot {\vec {e}}_{2}\geqslant 0 \end{aligned}$$

ensures that the cell and the substrate do not interpenetrate (Wriggers 2006).

## Electrostatic interactions are not sufficient for endothelial cell spreading

### Experimental evidence

In a multi-physics framework, *surface tractions* can be due to non-mechanical interactions: cell-to-cell and/or cell-to-ECM adhesion forces are as such.

Cell–cell and cell–ECM adhesion are receptor-mediated processes. The affinity of specific receptors modulates the adhesive forces, which may have a low electrostatic short range and a chemical receptor–ligand nature. A vast literature (Jacobs et al. 2013; Israelachvili 2011; Milo and Phillips 2016) has been devoted to quantifying these interaction forces. During the first phase of extravasation, EPCs interact with the luminal surface of blood vessels that is a carbohydrate-coated surface, consisting of adsorbed glycoproteins and membrane-bound proteoglycans, collectively referred to as the endothelial glycocalyx. The thickness of the glycocalyx^1^ is on the order of 400–500 nm in vivo and ranges from 29 to 118 nm in vitro (Chappell et al. 2009). This initial EPC/EC interaction is mediated by electrostatic interactions, which are essential for cell arrest and allow the recruitment and engagement of receptors that eventually support the cell adhesion.

**Fig. 2:**
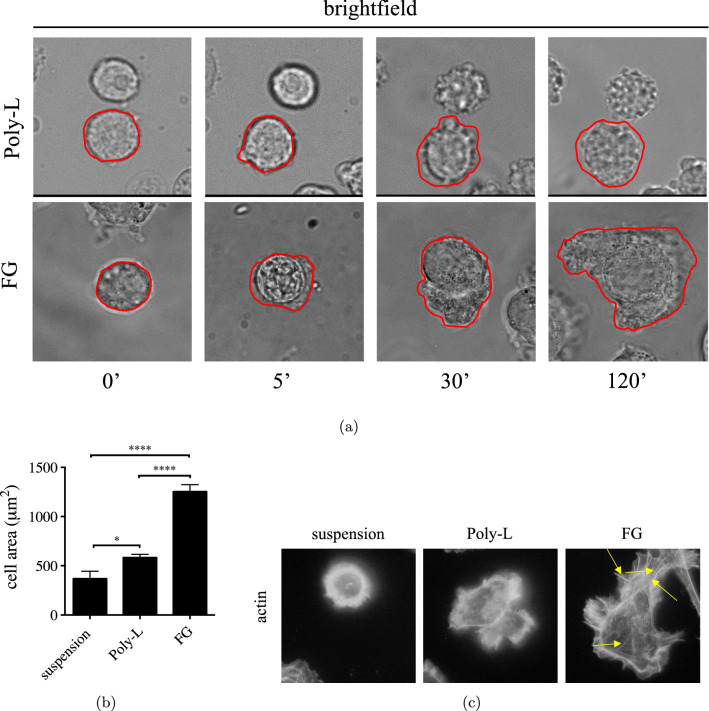
HUVECs seeded on different ECMs. HUVEC adhesion on different ECM. **a** Visualization of cell adhesion over time on Poly-L and FG; **b** quantification of cell area (${\mathrm{\mu m^{2}}}$) of HUVECs in suspension or seeded on FG or Poly-L at 2 h. Areas were measured by the ImageJ measurement tool. Results are expressed as mean ± SEM (40–60 cells/for each experimental condition); **c** actin cytoskeleton organization before and after 2 h of adhesion on FG or Poly-L. Yellow arrows show stress fibers

To assess if electrostatic forces drive the process of extravasation of EPCs, we investigated *in vitro* EC adhesion and spreading in the absence or in the presence of cytoskeleton remodeling. To this aim, ECs were plated on fibrinogen (FG) or on a positively charged synthetic polymer of L-lysine and D-lysine (Poly-L) and analyzed over time—see Fig. 2.

We demonstrated in Urbinati et al. (2012) that ECs attach on Poly-L without involving integrin receptors, focal adhesion formation, and cytoskeleton organization. Poly-L is less adhesive with respect to FG and about 50% of cells were removed with a saline buffer wash. In a new set of experiments, in this paper, we analyzed by time lapse videomicroscopy the timing of EC adhesion on FG and Poly-L. Figure 2a shows cells at different times of adhesion. ECs plated on FG rapidly attach to ECM and completely spread in 2 h, while EC is not able to spread on Poly-L. In Fig. 2b, the cell areas were calculated by imaging analysis using the measurement tool of ImageJ by drawing the contour of the cells. The lack of spreading on Poly-L and the estimation of the contact area were supported by the analysis of the actin cytoskeleton probed by phalloidin. Figure 2c clearly shows the different actin organizations in cells adherent on different matrices. Stress fibers and cortical cytoskeleton are formed in FG-adherent cells, while only the cortical actin is visible in suspension or in Poly-L adherent cells.

### Mechano-biological models

Short-range, noncovalent interactions that occur between one receptor and its ligand have been analyzed following two approaches available in the literature (Golestaneh and Nadler 2016; Ronan et al. 2014). According to (Golestaneh and Nadler 2016), at a given instant, the binding force per unit area in the current configuration is

3 $$\begin{aligned} \vec{t}(\vec{x}) = p_{N} \vec{e}_{2} = & - {\mathcal{C}}\left( {Kg_{N} \left( {\vec{x}} \right) + 1} \right)\left[ {\left( {Kg_{N} \left( {\vec{x}} \right) + 1} \right)^{2} + 1} \right]g_{N} \left( {\vec{x}} \right)^{{ - 5}} \\ & \,\exp ( - 2Kg_{N} \left( {\vec{x}} \right))\rho _{{rl}} \vec{e}_{2} , \\ \end{aligned}$$

where $\rho _{rl}$ is the minimum concentration of receptors and ligands at location $\vec {x}$, ${\mathcal {C}}$ is the number of weak noncovalent bonds which form the interaction between one receptor and one ligand, ${{\textit{g}_N}}$ has been defined in (2) and *K* is the inverse of the Debye length. We assume that ${\mathcal {C}}= 1.17 \times 10^{-7}$ ${\textrm{fN}} \mu {\textrm{m}}^{-5}$, $K=1$ provided in Golestaneh and Nadler (2016) apply to the problem at hand, too. Parameter $\rho _{rl}$ in Golestaneh and Nadler (2016) amounts at $10^{5}$ receptors per $\mu {\textrm{m}}^2$, severely higher than the concentration of species measured in Damioli et al. (2017). Such a parameter should be considered as a constant only if it refers to *all receptors on the membrane*, which is questionable. The protein transport affects the amount $\rho _{rl}$ and couples mechanical deformation in the bulk and chemo-transport processes on the membrane.

**Fig. 3:**
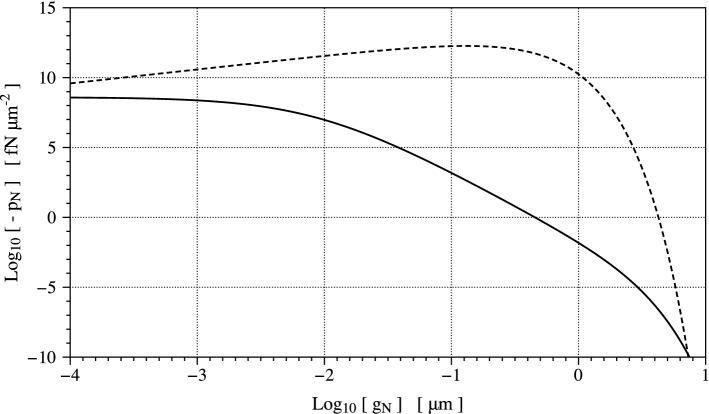
Comparison between binding forces on the membrane per unit area. The evolution of the Neumann electrostatic attractive tractions is depicted in a Log-Log plot with respect to the minimum distance $g_N$. Continuous line refers to Eq. (3) adopted in Golestaneh and Nadler (2016), whereas the dashed line refers to Eq. (4) used in Ronan et al. (2014)

The resulting Neumann electrostatic attractive tractions are plotted with a continuous line in Fig. 3. These forces decrease when the distance between receptors and ligands grows and are quite high at a strictly positive lower bound $h_0$ depicted as the gap between the cell and the substrate at contact. Authors in Golestaneh and Nadler (2016) suggest $h_0 = 9.0$ nm. Attractive forces decay rapidly and at a distance of ${\mathrm{0.5}}$ $\mu {\textrm{m}}$ they amount to a few ${\textrm{fN}} / \mu m^2$. Their range being so short, it is unlikely that those forces promote the cell spreading unless the characteristic size of the cell becomes very small (indeed, authors in Golestaneh and Nadler (2016) considered a cell with radius ${\mathrm{12.5}}$ ${\textrm{nm}}$, three orders of magnitude smaller than the measured radius of an EC in suspension - about ${\textrm{10}}$ ${\mathrm{\mu }}m$).

Attractive forces used in Ronan et al. (2014) to allow a cell to (partially) spread read

4 $$\begin{aligned} \vec {t}(\vec {x}) = p_N {\vec {e}}_{2}= - Q \; \frac{{{\textit{g}_N{\left( \vec {x}\right) }}}}{\delta _p} \, \exp (- \frac{{{\textit{g}_N{\left( \vec {x}\right) }}}}{\delta _p} ) \; {\vec {e}}_{2}\end{aligned}$$

with parameters $Q= \mathrm 5\cdot 10^7 fN/{\mu m}^2$, $\delta _p=$ 0.13 ${\mathrm{\mu }}m$, respectively. To promote cell spreading, these attraction forces result in four orders of magnitude higher than their counterpart in Eq. (3)—see the dashed line in Fig. 3. There is apparently no justification in the literature for such a huge value of the ligand-receptor binding electrostatic interactions acting on such a long-range extent.

In conclusion, electrostatic tractions can be invoked as responsible for the isotropic early stage of cell adhesion, which is essentially independent on cytoskeleton remodeling (Liu et al. 2007). Surface forces may drive the post-adhesion processes only for *nanoparticles* — as the ones considered in Golestaneh and Nadler (2016); Sohail et al. (2013). In ECs, electrostatic interactions are followed by the formation of membrane protrusion. As a cell begins to flatten against the substrate, it forms additional bonds, creates new focal adhesions, and rearranges its cytoskeleton to form actin filaments and bundles. Spreading of EC thus is a result of extensional and contractile forces exerted by the cytoskeleton machinery (Reinhart-King et al. 2005).

### Consequences on the mechanical modeling

Whereas surface tractions shall be estimated with high accuracy in modeling cell migration, since cells detach their focal adhesions in order to move (Doyle et al. 2021), the process of spreading is less sensitive to the values of binding forces, because it does not entail focal adhesions disruption. Therefore, cell spreading on a substrate can be to a first approximation modeled with contact mechanics, disregarding electrostatic tractions in view of the very short range of those interactions and accounting for pseudopodia extension and cytoskeletal contractility that induce spreading.

We modeled the glass-made $\mu$slide as a rigid obstacle, fixed in time. Hence, the global search for contact and the set-up of kinematical relations required by the contact constraints are straightforward. Contact occurs when ${{\textit{g}_N}}=0$. Such a condition defines the subpart $\partial ^C \Omega (t)$ in Fig. 1c. Tractions $\vec {t}_{slide}$ at all points $\vec {x}_{slide} \in \partial ^C \Omega (t)$ have normal component $p_N$ and the Hertz–Signorini–Moreau linear complementarity conditions for frictionless contact read

5 $$g_{N} \ge 0,\qquad p_{N} \le 0,\qquad p_{N} \;g_{N} = 0.{\text{ }}$$

The most appropriate description of tangential contact forces during the adhesion phase is rather unclear. We assume that the contact is frictionless, allowing a sliding motion between the cell and the substrate. Such an assumption is justified by the chance that a new complex can be formed by a previously ligand-engaged receptor that detaches and binds a nearby free ligand (Ronan et al. 2014).

Once the adhesion phase has completed, external cues trigger the pseudopodia-driven protrusion machinery (F-actin polymerization, actin branching, filamin cross-linking, integrin binding), driving the polymerization and reorganization of the cytoskeleton. We simulate these processes through the setup and evolution in time of bulk forces, oriented axis-symmetrically, surrogating the constitutive laws that link ${\varvec{F}}$ to the first Piola stress tensor ${\varvec{P}}$.

## Relocation of VEGFR2 on an advecting cell

**Fig. 4:**
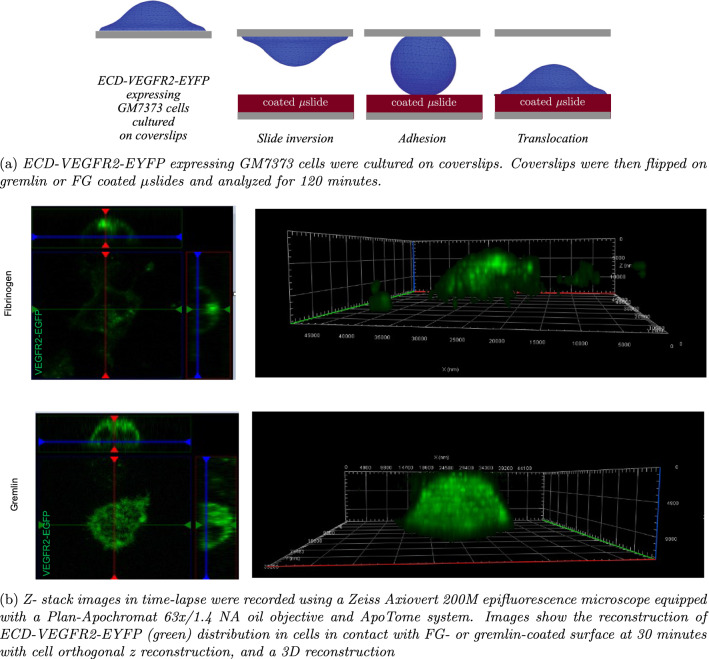
Experimental investigation on the relocation of VEGFR2 on an advecting cell

**Fig. 5:**
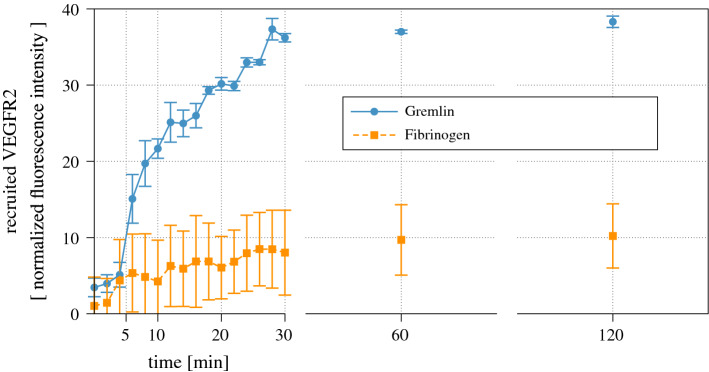
Quantification of normalized fluorescence of ECD-VEGFR2-EYFP on FG(orange line)- or Gremlin (blue line)-coated surfaces during the cell adhesion described in Fig. 4 (Mean SEM, *n* = 4)

### VEGFR2 is recruited at the basal portion of endothelial cells

To follow in time the relocation of VEGFR2, GM7373 cells were first transfected^2^ and grown on glass coverslips - Fig. 4a. Adhered cells were flipped upside-down afterward on gremlin- or FG-coated $\mu$slides. The concurrent geometrical evolution and VEGFR2 relocation were recorded for 2 h in time-lapse videomicroscopy - Fig. 4b. In this timespan, adhered cells moved from the glass coverslip to the protein-coated substratum, ultimately detaching from the upper coverslip. VEGFR2 relocated to the membrane portion in contact with immobilized gremlin already 6–8 min after the interaction with the substratum; in contrast, the adhesion of ECs to FG was not associated with a significant VEGFR2 polarization.

### Mechanical modeling

We will name *translocation* the aforementioned deformation process. It mimics cell migration and involves the already organized cytoskeleton. We will rather adopt the terminology *spreading* when the cytoskeleton is not initially organized. All events depicted in Fig. 4a have been accounted for in the numerical simulation of the in vitro experiment.

The translocation of an EC has been captured with a finite deformation model, detailed in Serpelloni et al. (2021, 2022). The *mechanical boundary value problem for adhesion and translocation* includes the balance of momentum equations, the contact constraint (5), and the solvability conditions. Mass and momentum balance equations are constitutively coupled, in the sense that stresses and fluxes are related to concentrations and displacements. Although the overall picture is rather clear, the response, polymerization, shape, and time evolution of bundles of filaments have not been captured by comprehensive models at the “macroscopic” scale by means of suitable free energies. Abundant research is still required to gain predicting computational capabilities in the reorganization of the cytoskeleton. For this sake, in order to simulate the relocation of proteins on the advecting membrane, we neglected the contractile active response of the cell and made use of rubber visco-elastic passive constitutive models for the entire cell during translocation.

Computationally, one writes the weak form of the boundary value problem in terms of displacements $\vec {u}_j( \vec {X})$ in the reference configuration

6 $$\begin{aligned}{} & {} \int _{\Omega _R} \delta {\varvec{F}} _i (\vec {X}) \,: \, {\varvec{P}} ( \vec {u}_j( \vec {X}) ) \, {\textrm{d}}V_R - \int _{\Omega _R} \delta \vec {u}_i( \vec {X}) \, \cdot \, \vec {b}_R( \vec {X}) \, {\textrm{d}}V_R \nonumber \\{} & {} \qquad - \int _{\partial ^N \Omega } \delta \vec {u}_i( \vec {X}) \, \cdot \, {\varvec{P}} ( \vec {u}_j( \vec {X}) ) \vec {n}_R \, {\textrm{d}}a = 0 \end{aligned}$$

with $\vec {b}_R( \vec {X})$ denoting referential bulk forces, ${\varvec{P}}$ the first Piola stress tensor, ${\varvec{F}}$ the deformation gradient, $\vec {n}_R$ the normal vector to the Neumann surface $\partial ^N \Omega$ in the reference configuration. A strategy to numerically deal with the contact constraints must be selected: among several possible algorithms, we implemented two classical active set methodologies, the Lagrange multiplier method (Wriggers 2006) and the primal-dual active set strategy (Hüeber and Wohlmuth 2005). Note also that frictionless contact does not allow unique solvability unless the rigid body motion in the plane of the $\mu$slide is removed. To this aim, the average displacement in the $\mu$slide plane ${\vec {e} }_{1}\times {\vec {e}}_{3}$ and rotations around axis ${\vec {e}}_{2}$ (see Fig. 1c) is a priori imposed to vanish.

Our simulations exploit iterative approximation schemes of staggered nature. On that account, the numerical solution of the mechanical problem (6) is achieved first, whereas the numerical approximation of the relocation of proteins on the membrane follows, eventually using different time discretizations.

### Chemical kinetics and transport modeling

The association and formation of a protein complex can be chemically denoted as

7 $$\begin{aligned} {\textrm{R}}+ {\textrm{L}} \mathop {\rightleftarrows }\limits _{k_b}^{k_f} {\textrm{C}} \end{aligned}$$

where ${\textrm{R}}$ and ${\textrm{L}}$ are the Receptors and Ligands free proteins and ${\textrm{C}}$ is the final complex (Bell 1978). Henceforth, we will add the subscripts ${}_{\textrm{R}}$, ${}_{\textrm{L}}$, and ${}_{\textrm{C}}$ to quantities associated with Receptor, Ligand, and Complex, respectively (as for $c_R$, $c_L$, and $c_C$ which denote the concentration of Receptors, Ligands, and Complexes). Coefficients $k_f$ and $k_b$ are the kinetic constants of the forward and backward reactions, respectively.

The transport-mechanics formulation (6) and the reaction (7) will be conveniently used to simulate the relocation of VEGFR2, driven by their specific ligands, on a plasma membrane $\partial \Omega (t)$. The rate of reaction (7), denoted with $w^{(7)}$and measured in ${\textrm{mol}} \, {m^{-2}s^{-1}}$, quantifies the net formation of ${\textrm{C}}$ on the advecting membrane as the difference between the forward and backward reactions.

The kinetics of reaction (7) is modeled as for ideal systems via the law of mass action (De Groot and Mazur 1984), which writes

8 $$\begin{aligned} w^{(7)}_R= k_{f_R} \,\frac{\vartheta _{\textrm{L}}}{(1- \vartheta _{\textrm{L}})} \,\frac{\vartheta _{\textrm{R}}}{(1- \vartheta _{\textrm{R}})} - \, k_{b_R} \, \frac{\vartheta _{\textrm{C}}}{(1- \vartheta _{\textrm{C}})} \end{aligned}$$

in the reference configuration. The ratios $\vartheta _{\textrm{R}}= c_{{\textrm{R}}_R}/ {c_{{\textrm{R}}_R}^{max}}$, $\vartheta _{\textrm{L}}= c_{{\textrm{L}}_R}/ {c_{{\textrm{L}}_R}^{max}}$, and $\vartheta _{\textrm{C}}= c_{{\textrm{C}}_R}/ {c_{{\textrm{C}}_R}^{max}}$ account for the saturation limits ${c_{{\textrm{R}}_R}^{max}}$, ${c_{{\textrm{L}}_R}^{max}}$, and ${c_{{\textrm{C}}_R}^{max}}$ of the species involved in the reaction (7). As clarified in Serpelloni et al. (2021), the invariance of $\vartheta _{\textrm{R}}$, $\vartheta _{\textrm{L}}$, and $\vartheta _{\textrm{C}}$ with the configuration implies that the forward and backward “constants,” which encompass the dimensionality of $w^{(7)}({\vec {x}}, t)$, transform contravariantly into $k_{f_R}$ and $k_{b_R}$ . In the described experimental setup, ligands are prevented to flow along the substrate: given that complex molecules result from the interaction with immobile ligands, they are macroscopically steady as well, i.e.,

9 $$\begin{aligned} \vec {h}_{\textrm{L}} = \vec {h}_{\textrm{C}} = \vec {0} \; {,} \end{aligned}$$

where $\vec {h}_{\mathrm{\beta }}$ denotes the mass flux of a generic species $\beta$ in current configuration. Assuming that equilibrium for the reaction (7) holds, the law of mass action (8) reads

10a $$\begin{aligned} \dfrac{k_{f}}{ k_{b}} = \dfrac{k_{f_R}}{ k_{b_R}} = \, \, \frac{\vartheta _{{\textrm{C}}_{R}}}{(1- \vartheta _{{\textrm{C}}_{R}})}\frac{(1-\vartheta _{{\textrm{L}}_{R}})}{\vartheta _{{\textrm{L}}_{R}}} \,\frac{(1- \vartheta _{{\textrm{R}}_{R}})}{\vartheta _{{\textrm{R}}_{R}}} =K_{\textrm{eq}}^{(7)}\;, \end{aligned}$$

where $K_\textrm{eq}^{(7)}$ is the so-called invariant equilibrium constant. It can be expressed as a function of the ratio between the standard Gibbs free energy $\Delta G^{0}$ and the product between the universal gas constant $\mathcal {R}$ and the absolute temperature *T* as

11 $$\begin{aligned} K_\textrm{eq}^{(7)}=\exp {\left( -\dfrac{\Delta G^{0}}{\mathrm{\mathcal {R}} T}\right) } \end{aligned}$$

see (Salvadori et al. 2018) for details. By explicitly accounting for reaction kinetics, the local form of the mass balance specifies as follows

12a $$\begin{aligned}&\frac{ \partial {c_{\textrm{R}_R}} }{ \partial t} + \, \textrm{Div}_{{{\mathcal {P}}}_R} \left[ \, { { \vec {h}}_{\textrm{R}_R} } \, \right] + \, w^{(7)}_R \; = 0 \; , \end{aligned}$$

12b $$\begin{aligned}&\frac{ \partial {c_{\textrm{L}_R}} }{ \partial t} + \, w^{(7)}_R \; = 0 \; , \end{aligned}$$

12c $$\begin{aligned}&\frac{ \partial {c_{\textrm{C}_R}} }{ \partial t} - \, w^{(7)}_R \; = 0 \; . \end{aligned}$$

Equation (12a) is defined on the membrane surface $\partial \Omega _R$, where the receptors flow. Equation (12b) is rather defined on the lower $\mu$slide, where ligands stand. Finally, Eq. (12c) is defined in the contact zone between the cell and the slide, where reaction (7) takes place. It is convenient to rephrase Eq. (12b) in terms of the “ligands *available* for the reaction” in place of the “ligands adsorbed on the $\mu$slide,” since this point of view corresponds to the picture of tight receptor–ligand bond as a set of weak noncovalent physical interactions (Alberts 2002). A supply function $s_{\textrm{L}_R}$, which vanishes at long ranges and rapidly reaches the *maximal concentration of ligands available for the reaction* at short distances, is defined to this purpose and Eq. (12b) is rewritten as follows

13 $$\begin{aligned} \frac{ \partial {c_{\textrm{L}_R}} }{ \partial t} + \, w^{(7)}_R \; = s_{\textrm{L}_R} \; . \end{aligned}$$

The ligand supply $s_{\textrm{L}_R}({\vec {X}}, t)$ seems to be logically related to the gap function ${{\textit{g}_N}}$ and to a lag in time that depicts the chemical kinetics of the binding–unbinding reaction (7). In this form, all three equations (12a), (12c), (13) can be written on the membrane $\vec {X} \in \partial \Omega _R$.

It will be further assumed that the time scale of the chemical reaction is much faster than other processes. Therefore, the concentrations of species are governed by thermodynamic equilibrium at all times. The concentration of complex $c_{\textrm{C}_R}$ relates then to the others by

14 $$\begin{aligned} {c_{\textrm{C}_R}} = \frac{ c^{ max}_\textrm{C} }{ c^{ max}_\textrm{R} \, c^{ max}_\textrm{L} } \; \frac{ K_\textrm{eq}^{(7)} }{ J \; | {\varvec{F}} ^{-T} \vec {n}_R | } \; c_{\textrm{R}_R} \, c_{\textrm{L}_R} \; , \end{aligned}$$

which emanates from the equation $w^{(7)}=0$ and is consistent with the assumptions made in Serpelloni et al. (2021) on how saturations transform.

Fickian thermodynamic restrictions linearly correlate $\vec {h}_{\textrm{R}_R}$ to the gradient of its chemical potential in terms of concentrations (see (Serpelloni et al. 2021) for details)

15 $$\begin{aligned} \vec {h}_{\textrm{R}_R} = - \textrm{D} \!\! | \,_\textrm{R} \, \textrm{Grad}_{{{\mathcal {P}}}_R} \left[ \, { c_{\textrm{R}_R} } \, \right] \; , \end{aligned}$$

where $\textrm{D} \!\! | \,_\textrm{R}$ is the receptor *diffusivity*.

In conclusion, exploiting identities (14) and (15), the two concentrations $c_{\textrm{R}_R}$ and $c_{\textrm{L}_R}$ fully describe the problem in the assumption of infinitely fast kinetics, whereas the concentration of the complex can be deduced a posteriori. The two balance equations

16a $$\begin{aligned}&\frac{ \partial {c_{\textrm{R}_R}} }{ \partial t} - \, \frac{ \partial {c_{\textrm{L}_R}} }{ \partial t} - \, \textrm{Div}_{{{\mathcal {P}}}_R} \left[ \, { \textrm{D} \!\! | \,_\textrm{R} \, \textrm{Grad}_{\mathcal{P}_R} \left[ \, { c_{\textrm{R}_R} } \, \right] } \, \right] \; = - s_{\textrm{L}_R} \; , \end{aligned}$$

16b $$\begin{aligned}&\frac{ \partial {c_{\textrm{L}_R}} }{ \partial t} + \, \frac{ \partial {c_{\textrm{C}_R}} }{ \partial t} \; = s_{\textrm{L}_R} \; , \end{aligned}$$

govern the transport of receptors along the membrane at point $\vec {X} \in \partial \Omega _R$, provided that initial and boundary conditions are given.

An effective way of solving this system can be set up by noting that for the sum $c_{\textrm{S}_R} = c_{\textrm{C}_R} + c_{\textrm{L}_R}$ equation (16b) turns out to be an ordinary differential equation in time at point $\vec {X}$. By direct integration

17 $$\begin{aligned} c_{\textrm{S}_R} = \int _0^t \, s_{\textrm{L}_R} ( \vec {X}, \tau ) \, \textrm{d} \tau = S_{\textrm{L}_R} ( \vec {X}, t ) \; , \end{aligned}$$

assuming initial concentration of ligands and complex to be zero. Function $S_{\textrm{L}_R} ( \vec {X}, t )$ is, in view of the interpretation given to $s_{\textrm{L}_R} ( \vec {X}, \tau )$, the amount of ligands *conformationally available for the reaction* at time *t* and location $\vec {X}$, pulled back to the reference configuration $\partial \Omega _R$. Such an interpretation clarifies that the evolution in time of $S_{\textrm{L}_R} ( \vec {X}, t )$ can only be due to the gap function ${{\textit{g}_N}}(\vec {X}, t )$ and we may reasonably postulate

18 $$\begin{aligned} S_{\textrm{L}_R} ( \vec {X}, t ) = c_{\textrm{L}_R}^{av} \exp ( - \frac{ {{\textit{g}_N}}(\vec {X}, t ) }{ \ell _{chem}} ) \end{aligned}$$

based on the physics that has been described right after equation (13). In eq. (18), $\ell _{chem}>0$ is a *chemical length-scale* that tunes the amount of available ligands to the gap $g_N$ and, numerically, acquires the meaning of a regularization parameter, while $c_{\textrm{L}_R}^{av}$ is the maximum amount of available ligands on $\partial \Omega (t)$. When the dimensionless number ${{\textit{g}_N}}(\vec {X}, t ) / \ell _{chem}$ is sufficiently large then $S_{\textrm{L}_R} ( \vec {X}, t )$ becomes negligible and in fact no ligands are available on the membrane surface. On the other end, when ${{\textit{g}_N}}(\vec {X}, t )$ is zero and the membrane is in contact with the substrate, the amount of available ligands is maximal. Note that we are not capable to measure $c_{\textrm{L}_R}^{av}$ experimentally. In fact, molecules shall be in a specific geometrical configuration in order to interact with VEGFR2. Only a fraction, hardly quantifiable experimentally, of all ligands (the ones denoted as “available”) are properly arranged. Therefore, $c_\mathrm{L_R}^{ av}$ will be determined numerically in section 5, through a co-designed numerical (*in silico*) and experimental (*in vitro*) approach.

Defining the difference $c_{\textrm{D}_R} = c_{\textrm{R}_R} - c_{\textrm{L}_R}$ and neglecting the role of internalization or generation of proteins, the remaining governing equation becomes

19a $$\begin{aligned} \frac{ \partial {c_{\textrm{D}_R}} }{ \partial t} - \, \textrm{Div}_{ \Omega _R } \left[ \, { \textrm{D} \!\! | \,_\textrm{R} \, \textrm{Grad}_{ \Omega _R} \left[ \, { c_{\textrm{R}_R} } \, \right] } \, \right] \; = - s_{\textrm{L}_R} \; , \end{aligned}$$

to be solved under the constraint (14), that writes

19b $$\begin{aligned}{} & {} c_{\textrm{R}_R} ^2 (\vec {X}, t) + \left[ \alpha _R(\vec {X}, t) - c_{\textrm{D}_R} (\vec {X}, t) \right] \, c_{\textrm{R}_R}(\vec {X}, t) \nonumber \\{} & {} \qquad - \alpha _R(\vec {X}, t) \, \left[ S_{\textrm{L}_R} ( \vec {X}, t ) + c_{\textrm{D}_R}(\vec {X}, t) \right] \, = 0 \;, \end{aligned}$$

with

20 $$\begin{aligned} \alpha _R(\vec {X}, t) = \frac{ c^\mathrm{{max}}_\textrm{R} \, c^\mathrm{{max}}_\textrm{L} }{ c^\mathrm{{max}}_\textrm{C} } \; \frac{ J \; | {\varvec{F}} ^{-T} \vec {n}_R | }{ K_\textrm{eq}^{(7)} } \; . \end{aligned}$$

The initial value problem (19) will be solved for the unknown fields $c_{\textrm{D}_R}$, $c_{\textrm{R}_R}$.

The finite element approximation of the chemo-diffusive problem of VEGFR2 on the cell membrane stems from its weak form, obtained after multiplying Eq. (19a) by a suitable set of time independent test functions (expressed here with a superposed caret) and performing an integration upon the domain, exploiting Green’s formula with the aim of reducing the order of differentiation. It reads:

21 $$\begin{aligned}&\int _{{{\mathcal {P}}_R}} \hat{c}( \vec {X} ) \; \frac{ \partial c_{\textrm{D}_R} (\vec {X}, t ) }{ \partial t} \; \nonumber \\&\qquad + \textrm{Grad}_{{\mathcal {P}}_R} \left[ \, { \hat{c}( \vec {X} ) } \, \right] \cdot \; \textrm{D} \!\! | \,_\textrm{R} \, \textrm{Grad}_{{\mathcal {P}}_R} \left[ \, { c_{\textrm{R}_R} (\vec {X}, t ) } \, \right] \; \nonumber \\&\qquad + \hat{c}( \vec {X} ) \; s_{\textrm{L}_R} (\vec {X}, t ) \; \; \textrm{d} {{\mathcal {P}}_R} = 0\;. \end{aligned}$$

The weak form (21) naturally leads to a semi-discrete problem, by approximating the unknown fields $c_{\textrm{D}_R}$, $c_{\textrm{R}_R}$ as a product of separated variables (by means of spatial shape functions and nodal unknowns that depend solely on time)

22 $$\begin{aligned} c_{\textrm{D}_h}(\vec {X}, t ) = \varphi _{j} (\vec {X}) \; {c_{\textrm{D}_R}}_{j}(t) \; , \qquad c_{\textrm{R}_h}(\vec {X}, t ) = \varphi _{j} (\vec {X}) \; {c_{\textrm{R}_R}}_{j}(t) \; , \end{aligned}$$

where, for repeated indexes, the Einstein summation convention is taken.

To obtain a full discretization of the weak form (21), a uniform mesh for the time variable *t* has been taken. By defining $t_n = n \, \Delta t$ with $n = 0, 1,...$ and $\Delta t > 0$, we integrate the semi-discrete form of (21) in time in the generic interval $t_{n-1}, t_n$ to eventually get

23 $$\begin{aligned} \int _{{\mathcal {P}}_R}&\varphi _{i} (\vec {X}) \; \varphi _{j} (\vec {X}) \; \textrm{d} {{\mathcal {P}}_R} \; \left( {c_{\textrm{D}_R}}_{j}(t_n ) - {c_{\textrm{D}_R}}_{j}(t_{n-1} ) \right) \nonumber \\&+ \int _{{\mathcal {P}}_R} \textrm{Grad}_{{\mathcal {P}}_R} \left[ \, { \varphi _{i} (\vec {X}) } \, \right] \cdot \; \textrm{D} \!\! | \,_\textrm{R} \, \textrm{Grad}_{{\mathcal {P}}_R} \left[ \, { \varphi _{j} (\vec {X}) } \, \right] \; \textrm{d} {{\mathcal {P}}_R} \; \nonumber \\&\int _{t_{n-1}}^{t_n} {c_{\textrm{R}_R}}_{j}(t) \; \textrm{d} t \; + \nonumber \\&+ \int _{{\mathcal {P}}_R} \varphi _{i} (\vec {X}) \; \left( S_{\textrm{L}_R} ( \vec {X}, t_n ) - S_{\textrm{L}_R} ( \vec {X}, t_{n-1} ) \right) \; \; \textrm{d} {{\mathcal {P}}_R} = 0 \; . \end{aligned}$$

The Newton–Cotes quadrature formula

$$\begin{aligned} \; \int _{t_{n-1}}^{t_n} {c_{\textrm{R}_R}}_{j}(t) \textrm{d} t \sim \frac{\Delta t}{2} \left[ \; {c_{\textrm{R}_R}}_{j}(t_n ) + {c_{\textrm{R}_R}}_{j}(t_{n-1} ) \; \right] \end{aligned}$$

is adopted to approximate the integral in time. Finally, the constraint (19b) has been imposed numerically, either in $L_2$ sense or point-wise for ${c_{\textrm{D}_R}}_{j}(t)$, ${c_{\textrm{R}_R}}_{j}(t)$.

The finite element approximation (23) has been implemented exploiting deal.ii (Arndt et al. 2021), a high-performance computing open source, object oriented library (https://www.dealii.org/).

### An evolution of the model

FRAP analysis shows a peculiar feature of VEGFR2, which may require an evolution of the model described so far. A fraction of VEGFR2 is in fact retained in an immobile compartment (Grillo et al. 2021): It was estimated that nearly 23% of VEGFR2 is basically immobile at each material point of the cell membrane. At every point of the membrane, only 77% of ECD-VEGFR2-EYFP is supposed to be in a mobile form (Damioli et al. 2017).

To capture this event, the VEGFR2 molecules may be separated into two independent fractions with different motilities, named $\mathrm{R^i}$ (immobile) and $\mathrm{R^m}$ (mobile). The reaction (7) shall be split into two, as

24 $$\begin{aligned}&\mathrm{R^i}+ \textrm{L} \mathop {\rightleftarrows }\limits _{k_b}^{k_f} \mathrm{C^i} \; ,&\mathrm{R^m}+ \textrm{L} \mathop {\rightleftarrows }\limits _{k_b}^{k_f} \mathrm{C^m} \; . \end{aligned}$$

Because the chemical interaction exerted by immobile and mobile VEGFR2 is totally equivalent, the kinetic constants of the forward and backward reactions are the same. Balance equations (12) rewrite as follows:

25a $$\begin{aligned}&\dfrac{\partial c_{{\mathrm{R^i}}_R}}{\partial t} +w_{R}^{(24\textrm{i})}= 0 \; , \end{aligned}$$

25b $$\begin{aligned}&\dfrac{\partial c_{{\mathrm{R^m}}_R}}{\partial t}+ \textrm{Div}_{{\mathcal {P}}_R} \left[ \, {\vec {h}_{{\mathrm{R^m}}_R} } \, \right] +w_{R}^{(24\textrm{m})}= 0 \; , \end{aligned}$$

25c $$\begin{aligned}&\dfrac{\partial c_{\textrm{L}_{R}}}{\partial t}+w_{R}^{(24\textrm{i})}+w_{R}^{(24\textrm{m})}=s_{\textrm{L}_{R}} \; , \end{aligned}$$

25d $$\begin{aligned}&\dfrac{\partial c_{\textrm{C}_{R}}}{\partial t}=\dfrac{\partial c_{\mathrm{C^i}_{R}}}{\partial t}+\dfrac{\partial c_{\mathrm{C^m}_{R}}}{\partial t} \; , \end{aligned}$$

25e $$\begin{aligned}&\dfrac{\partial c_{\mathrm{C^i}_{R}}}{\partial t}=w_{R}^{(24\textrm{i})} \; , \end{aligned}$$

25f $$\begin{aligned}&\dfrac{\partial c_{\mathrm{C^m}_{R}}}{\partial t}=w_{R}^{(24\textrm{m})} \; . \end{aligned}$$

Specifications on the receptor–ligand interplay can be inherited

26 $$\begin{aligned}&c_{\mathrm{C^i}_{R}}=\dfrac{c_{{\mathrm{R^i}}_R}c_{\textrm{L}_{R}}}{\alpha _R} \; ,&c_{\mathrm{C^m}_{R}}=\dfrac{c_{{\mathrm{R^m}}_R}c_{\textrm{L}_{R}}}{\alpha _R} \; , \end{aligned}$$

together with Fick’s law

27 $$\begin{aligned} \vec {h}_{\mathrm{R^m}_R} = - \textrm{D} \!\! | \,_\textrm{R} \, \textrm{Grad}_{\mathcal{P}_R} \left[ \, { c_{\mathrm{R^m}_R} } \, \right] \; , \end{aligned}$$

where $\textrm{D} \!\! | \,_\textrm{R}$ is the receptor *diffusivity*. The governing equations (16) thus will be replaced by:

28a $$\begin{aligned}&\dfrac{\partial c_{{\mathrm{R^i}}_R}}{\partial t} +\dfrac{\partial c_{\mathrm{C^i}_{R}}}{\partial t}=s_{{\mathrm{R^i}}_R} \; , \end{aligned}$$

28b $$\begin{aligned}&\dfrac{\partial c_{{\mathrm{R^m}}_R}}{\partial t}+ \textrm{Div}_{{\mathcal {P}}_R} \left[ \, {\vec {h}_{{\mathrm{R^m}}_R} } \, \right] +\dfrac{\partial c_{\mathrm{C^m}_{R}}}{\partial t}=s_{{\mathrm{R^m}}_R} \; , \end{aligned}$$

28c $$\begin{aligned}&\dfrac{\partial c_{\textrm{L}_{R}}}{\partial t}+\dfrac{\partial c_{\mathrm{C^i}_{R}}}{\partial t}+\dfrac{\partial c_{\mathrm{C^m}_{R}}}{\partial t}=s_{\textrm{L}_{R}} \; , \end{aligned}$$

28d $$\begin{aligned}&\dfrac{\partial c_{\textrm{C}_{R}}}{\partial t}=\dfrac{\partial c_{\mathrm{C^i}_{R}}}{\partial t}+\dfrac{\partial c_{\mathrm{C^m}_{R}}}{\partial t} \; . \end{aligned}$$

## Simulations

Since this work focuses on the relocation of VEGFR2, which is not primarily involved in the cytoskeleton reorganization, we did not explicitly account for focal adhesions in the numerical simulations. Accordingly, the transport of proteins along the membrane does not influence the mechanical deformation and the staggered algorithm that has been implemented does not require prediction and correction phases (Martins et al. 2017): The mechanical deformation within a given time step is followed by transport coupled with receptors–ligands binding on the updated membrane configuration.

In the numerical simulation, different time-steps $\Delta t$ have been used in the three stages (attachment, translocation, diffusion) of the in silico experiment: $0.5\ \text {s}$ for the chemically dominated attachment phase, $0.1\ \text {s}$ during the mechanical translocation—chemomechanically dominated, and $0.05\ \text {s}$ afterward ( diffusion dominated ). Furthermore, being conscious that the time scale of the bulk and membrane processes are different, a sub-incrementation strategy for the numerical solution of the chemo-diffusive problem on the cell surface has been adopted. The chemo-diffusive problem has been solved with a time step a hundred times smaller than the “mechanical” time step.

**Fig. 6:**
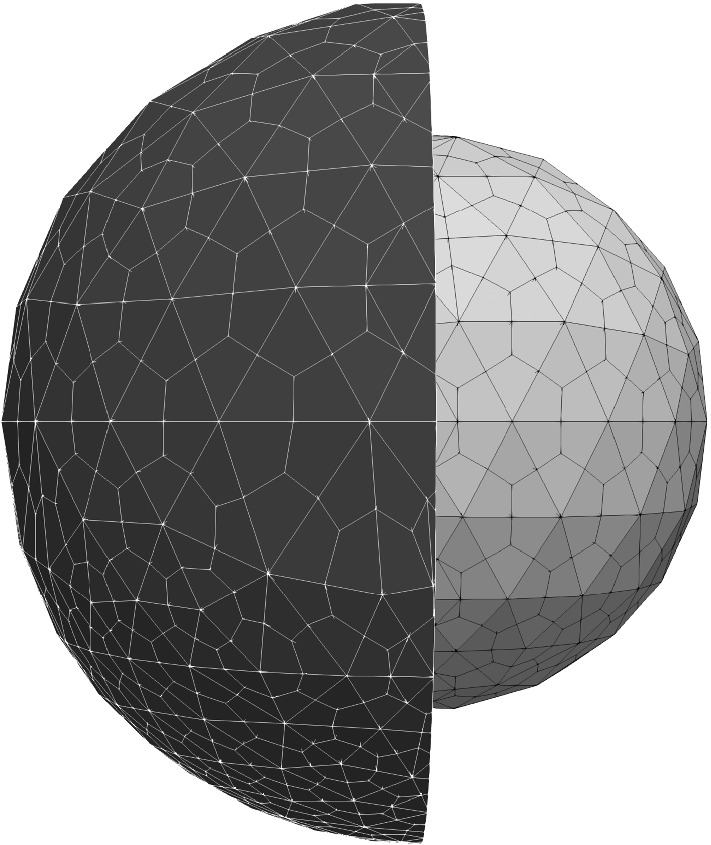
An unstructured grid of the reference configuration $\Omega _R$ with 10080 hexahedral elements, biased toward the cell bottom, which has been used in the simulations. The nucleus and the cytoplasm surface mesh have been highlighted: 1818 faces discretize the geometry of the plasma membrane

Several unstructured grids have been used for convergence tests. Numerical outcomes that will be described later have been obtained with the tessellation depicted in Fig. 6.

### Model calibration

**Table 2:** Set of constitutive parameters (bulk modulus $\kappa$ and shear modulus *G*) used in finite element simulations in McGarry and Prendergast (2004); Heyden and Ortiz (2017) as well as identified in this work through co-designed experiments and simulations

	Note	$\kappa$ [Pa]	*G* [Pa]	Ref.
Cytoplasm	Cancerous	$39.733 \times 10^{3}$	$1.664 \times 10^{3}$	Heyden and Ortiz (2017)
	Healthy	$71.520 \times 10^{3}$	$2.995 \times 10^{3}$	Heyden and Ortiz (2017)
	Healthy	128.205	36.49	McGarry and Prendergast (2004)
	Healthy	12.82	3.65	*This work*
Nucleus	Cancerous	$239.989 \times 10^{3}$	$9.664 \times 10^{3}$	Heyden and Ortiz (2017)
	Healthy	$431.98 \times 10^{3}$	$17.395 \times 10^{3}$	Heyden and Ortiz (2017)
	Healthy	512.821	145.985	McGarry and Prendergast (2004)
	Healthy	25.641	7.299	*This work*

**Mechanical constitutive parameters** - Heyden and Ortiz (2017) studied the material response of live metastatic cancer cells via the method of oncotripsy. Investigating the influence of viscoelasticity, two different sets of material parameters were considered, either in healthy or cancerous cells. The elasticity of the different cell constituents was modeled by means of a Mooney–Rivlin-type strain-energy density, leading to the material parameters in Table 2. In an earlier work, McGarry and Prendergast (2004); Guilak et al. (2000) proposed a three-dimensional FEM model of an adherent eukaryotic cell, treating the cytoplasm and nucleus as linear elastic and isotropic continua. The elastic modulus of the cytoplasm was chosen as 100 Pa, while the nucleus was chosen as 400 Pa, four times stiffer than the cytoplasm, as reported in Table 2.

**Fig. 7:**
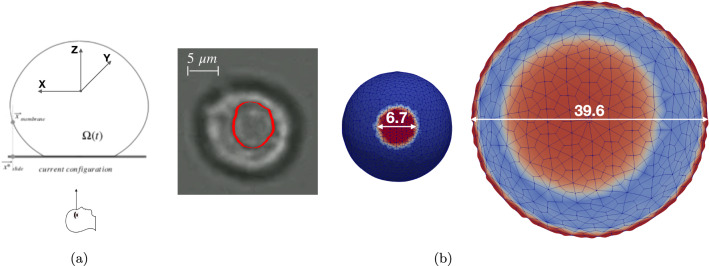
The cell contact area: **a** Notation; **b** the experimental evidence: the inner ring can be interpreted as the boundary of the contact area of ECs on Poly-Lysine; simulations at the end of the adhesion stage (diameter $6.7\ \mu m$, CA $35.3\ \mu m^2$) and at the end of the mechanical translocation phase (diameter $39.6\ \mu m$, CA $1232\ \mu m^2$)

We used a hyper-elastic Regularized Neo-Hookean formulation for the nucleus and the cytoplasm and estimated the shear modulus using data of ECs adhering to Poly-L. In these experiments a stable, although weak, attachment of the ECs on the $\mu$slide arose, comparable to the one at the early stage of adhesion on FG. An early contact area (CA) of approximately $38.5\ \mu \mathrm m^2$ was observed, as it can be deduced from Fig. 7b.

The numerical simulations of attachment unveiled an optimal value for the shear modulus in the order of 3.6 Pa. At the end of the adhesion phase, in fact, the contact area was roughly equal to $35.3\ \mathrm \mu m^2$ — see Fig. 7a. The sensitivity of the bulk modulus appears to be less relevant: We noticed that a factor 4 between G and $\kappa$ as in McGarry and Prendergast is appropriate.

**Transport constitutive parameters ** - By means of surface plasmon resonance, we estimated in Maiolo et al. (2012) the value of $c_{\textrm{L}_R}^{max}= 16000\ \mathrm{mol / \mu m^{2}}$ and the standard Gibbs free energy $\varDelta G_0=-32949.0\ \textrm{J}/{mol}$. The equilibrium constant of reaction (7) descends $K_\textrm{eq}^{(7} = 354058.32$. The receptor diffusivity $\textrm{D} \!\! | \,_\textrm{R}=0.198$ $\mathrm{\mu m^2}{s^{-1}}$ was experimentally assessed in Damioli et al. (2017) through Fluorescence Recovery After Photobleaching (FRAP). It has been set $c^{ max}_\textrm{R} = c^{ max}_\textrm{C}$. The total number of molecules of VEGFR2 on the plasma membrane of an EC is taken equal to 24000 (maintained constant for all the time of the simulations, i.e., no internalization/exposure of receptors from/on the cell membrane is allowed), providing an initial concentration equal to $19.1\ \mathrm{molecules /\mu m^{2}}$, corresponding to a homogeneous distribution of receptors on the surface of a spheric cell in suspension with radius $r=10\ \mathrm \mu m$ (Damioli et al. 2017; Salvadori et al. 2018). Finally, the total number of available gremlin that coats the substrate $S_{\textrm{L}_R} ( \vec {X}, t )$ cannot be experimentally deduced a priori, because not all molecules are in the ideal geometrical configuration to interact with VEGFR2. These considerations prelude to the calibration of the concentration of available ligands in the substrate from the in silico analysis: $c_{\textrm{L}_R}^{av}$ has been found to be equal to $90\ \textrm{molecules}/{\mu m^2}$. Material parameters and data required by the numerical simulations have been collected in Table 3.

**Table 3:** Material parameters and data required by the numerical simulations

Parameter	Symbol	Value	Units
Maximum amount of available ligands on $\partial \Omega (t)$	$c_{\textrm{L}_R}^{av}$	90.00	$\left[ {\text {mol}}\cdot {\mu m^{-2}}\right]$
Ligands saturation limit	$c^\mathrm{{max}}_{\textrm{L}}$	$16\cdot 10^3$	$\left[ {\textrm{mol}} \cdot {\mu {\textrm{m}}^{-2}}\right]$
Equilibrium constant	$K^{(7}_{\textrm{eq}}$	354058.32	$\left[ -\right]$
Receptor diffusivity	$\textrm{D} \!\! | \,_R$	0.198	$\left[ {\mu {\textrm{m}}^{2}}\cdot s^{-1}\right]$
Bulk modulus cytoplasm	$\kappa$	12.82	$\left[ {\textrm{Pa}}\right]$
Shear modulus cytoplasm	G	3.65	$\left[ {\textrm{Pa}}\right]$
Bulk modulus nucleus	$\kappa$	25.641	$\left[ {\textrm{Pa}}\right]$
Shear modulus nucleus	G	7.299	$\left[ {\textrm{Pa}}\right]$
Cell initial radius	$r_0$	10.00	$\left[ {\mu {\textrm{m}}}\right]$
Cell initial surface	$A_0$	1256.64	$\left[ {\mu {\textrm{m}}^{2}}\right]$
Chemical length-scale	$\ell _\mathrm{{chem}}$	0.2	$\left[ {\mu {\textrm{m}}}\right]$

### Model validation and predictions

As stated earlier in the paper, we surrogate the cytoskeletal machinery with suitable bulk forces, axis-symmetrically oriented and capable to induce the cell translocation. Some authors describe those forces to be localized at the membrane: According to (Vernerey and Farsad 2014), protrusion forces act in the internal boundary of the membrane and are related to the integrin binding at the focal adhesion sites. The cell cortex was considered as an excitable system in Cooper et al. (2012), leading the cell to a zigzag crawling that was indeed experimentally observed. In Allena (2013), a decomposition of the deformation gradient was used to reproduce the cyclic phases of protrusion and contraction of the cell, which are tightly synchronized with the adhesion forces at the back and at the front of the cell.

In cellular motility, the filopodia and lamellipodia extension/retraction may convey a form of “active perception” for the cell, guiding the movement of VEGFR receptors rapidly through the local extracellular environment. In this way, the cell disposes of a quick mechanism that can rule the cell-response to the environmental conditions (Bentley and Chakravartula 2017).

Pseudopods are supposed to protrude in the direction of the most attractive location, as for the case of chemotaxis. This process is simulated by imposing bulk forces in the cytosol inversely proportional to the distance of the most attractive sensed location, tuned by means of a paraboloid filter function. This approach lacks the physical connection between the bulk forces and the actin polymerization, which is supposed to be captured chemo-mechanically by the swelling tensor ${\varvec{F}} ^s$ detailed in Serpelloni et al. (2021, 2022), Bonanno et al. 2023) and that will be accounted for in future research.

**Fig. 8:**
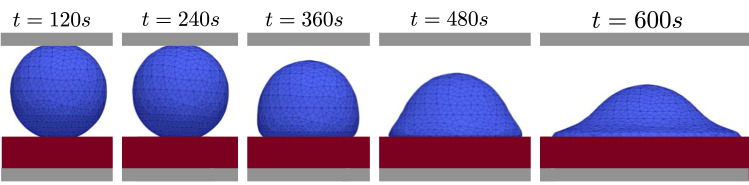
Cell-shape (axis-symmetry holds) at different instants

Some configurations during the simulations of translocation are depicted in Fig. 8. We took advantage of a few experimental data, the measured diameter $40\ \mu \mathrm m$ at the end of the translocation averaged on 50 cells and the duration of the mechanical adhesion (300 s) and translocation (600 s), to estimate the order of magnitude of the protrusion forces as 6.2 times the gravitational forces on the cell.

**Fig. 9:**
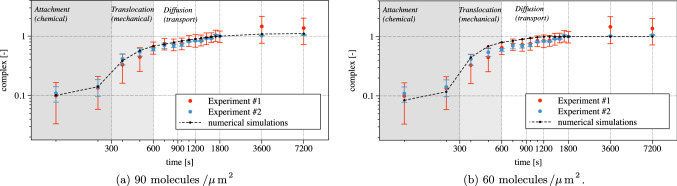
Numerical (dashed line) and experimental (white markers with error bars) evolution in time of the total amount of complex, normalized at 1800 s

Figure 9a compares the numerical and experimental amount of complex in time. In vitro experiments provide the total amount of fluorescence intensity measured at the basal side of the cell. Experimentally, concentration is estimated from fluorescence intensity. In silico experiments determine the quantity of molecules of complex generated on the cell-substrate contact area. Figure 9 compares in silico and in vitro outcomes: it’s a log–log plot, with time on the abscissa and complex concentration on the vertical axis. Both measures and numerical outcomes have been normalized to unity at time 1800 s, i.e., we normalized every in silico and in vitro data against the corresponding value at time 1800 s. Accordingly, the plotted concentration is dimensionless.

**Fig. 10:**
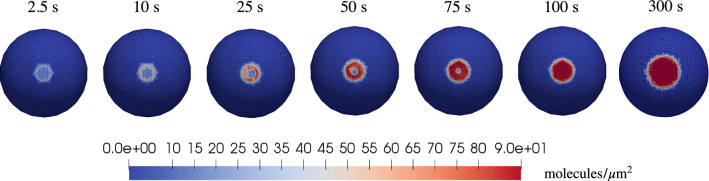
The coffee ring evolution in the first 100 s and the basal distribution of complexes at the end of the attachment stage. Note that, after 100 s, the concentration of complexes within the contact area is maximal (i.e., $90\ {molecules /\mu m^{2}}$)

Three regions are highlighted in Fig. 9a. The first 300 s (dark gray area) correspond to the contact and attachment phase. In this time span, complexes are generated by chemical interactions in the CA, and by the diffusion of receptors from the neighboring region. The reduction of free VEGFR2 is dominated by the chemical interaction between receptor and ligands as soon as the cell gets into contact with the substrate. The chemical reaction is further corroborated by a relevant relocation of free receptors through diffusion. In fact, after the first interaction between the cell surface and substrate, receptors become completely engaged because the number of available ligands on the substrate ($90\ \textrm{molecules}/{\mu m^2}$) is much higher than the amount of free receptors on the membrane ($19.1\ \mathrm{molecules /\mu m^{2}}$). The reduction of available VEGFR2 boosts the diffusion of free receptors from neighboring regions, due to Fick’s law. This phenomenon can be observed from the evolution of the complex concentration in Fig. 10. A “coffee ring” distribution of complexes is already evident at 10 s. Within such a ring, the concentration increases up to the ligand saturation, and the ring enlarges afterward, up to covering the entire CA 100 s after the first interaction. Note that this effect could not be seen in the small strains, two-dimensional simulations carried out in Damioli et al. (2017). At the end of the attachment phase, complexes are uniformly distributed throughout the CA.

**Fig. 11:**
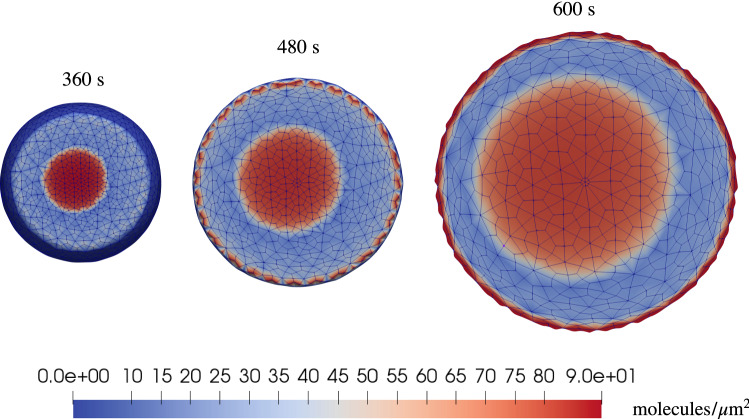
Basal distribution of complexes in the translocation stage

The second time span, highlighted in light gray between 300 and 600 s in Fig. 9a, corresponds to the mechanical translocation. More and more area becomes available for the reaction (7), which occurs very rapidly. This mechanical effect causes a remarkable increment of complexes, pinpointed by a steep tangent in the numerical curve. Moreover, the tendency of complexes to accumulate at the boundary of the cell-substrate contact area emerges quite clearly in Fig. 11. Here, a coffee ring appears on the border of the CA, as described in Damioli et al. (2017), owing to the timescale of the mechanics, which is faster than the free receptors diffusion on the membrane. Furthermore, a diffusive-like process for the complexes appears in the region between the adhesion area toward the coffee ring. In fact, complexes cannot flow, and the evolution of their concentration is ruled by the chemical reaction (7) together with the diffusion of free receptors in chemical equilibrium with complexes, which aim at occupying uniformly the CA during translocation.

**Fig. 12:**
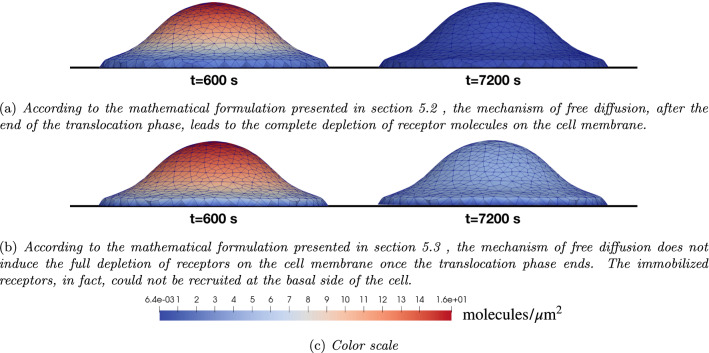
Diffusion of receptors after the translocation phase has been completed

Eventually, cell translocation ceases after 600 s. Diffusion of receptors on the membrane, which in first approximation can be considered as mechanically steady, carries on. This transport process, much slower than the other two mechanisms, is Brownian by nature and fueled by the concentration gradient. Free receptors move from the apical part of the cell toward the basal, where chemical interactions occur and decrease both free VEGFR2 and gremlin concentrations—see Fig. 12. Therefore, the final branch of the plot in Fig. 9a is transport-dominated and shows a low complex formation rate. The assumption of negligible internalization and exposure of VEGFR2 from/to the membrane seems thus to be acceptable: in reality, exposure and internalization compensate each other, being key processes during VEGFR2 activation in angiogenesis. Higher experimental uncertainties after 1800 s, highlighted by larger error bars, might be related to the slow synthesis process of new VEGFR2 in the bulk of the cell.

**Fig. 13:**
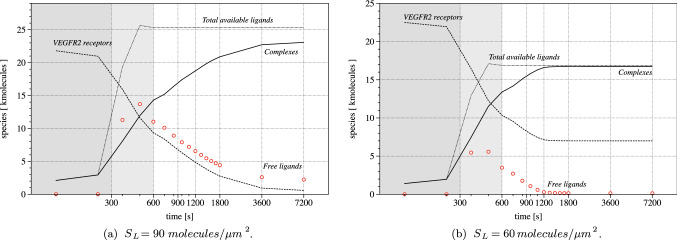
Evolution in time of the total amount of molecules of receptors (dashed curve), complexes (continuous), free (point markers) and available ligands (either free or bound) (dotted curve)

Integration of the concentrations over the membrane provides the total number of molecules at a given time. The evolution in time of such amount of complexes, free and available ligands, as well as unengaged VEGFR2, is plotted in Fig. 13a. We can verify that mass is conserved by checking the sum of the molecules of VEGFR2 and of complexes, which shall be constant in time in view of the stoichiometry of reaction (7). During the attachment phase, the total amount of ligands (free plus substrate bounded) perfectly overlaps the complexes. This evidence confirms that the chemo-diffusive phenomenon is dominant. It clearly emerges, instead, that during the mechanical translocation, from 300 s to 600 s, the amount of available ligands increases by a large extent. This phenomenon occurs because the time scale of the chemical reaction is faster than mechanics, which in turn is faster than transport: The latter cannot provide sufficient receptors to bind the large number of ligands that become available by the increment of CA granted by translocation. Receptors become fully engaged in complexes, whereas the chemo-diffusive process leads to the reduction of available ligands only later, basically at the end of the experiment.

### Validation against experimental data calls for an evolution of the model

Figures 12a and 13a show that at the end of the experimental time span, the amount of free receptor molecules predicted by the simulations is extremely small. On the contrary, fluorescence analysis shows that the residual amount of free receptors in the apical membrane after 7200 s is approximately $30\%$ of the initial concentration. The simplest way to cope with this biological fact is adjusting the number of available ligands, by reducing them from 90 to about $60\ \mathrm molecules/\mu m^2$. With such a tailoring, about the 30% of the total amount of VEGFR2 on the cell membrane results indeed to be unbound at the end of the numerical simulations—Fig. 13b. However, this approach modifies the complex formation curve, which now diverges from the experimental data—see Fig. 9b. This fact suggests that the observed discrepancy must have a different biological explanation, which can be captured using the governing equations (28) in place of Eqs. (16).

**Fig. 14:**
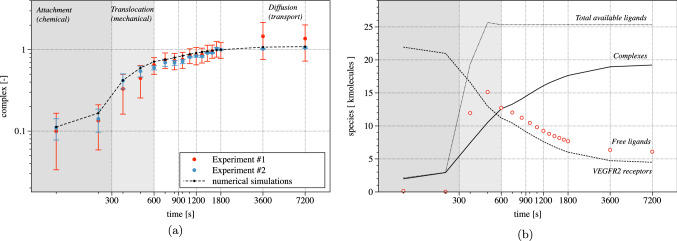
**a** Numerical (dashed line) and experimental (white markers with error bars) evolution in time of the total amount of complexes, normalized at 1800 s. **b** Evolution in time of the total amount of molecules of receptors (dashed curve), complexes (continuous), free (point markers) and available ligands (either free or bound) (dotted curve) accounting for the immobilized receptors

The splitting of receptors allows numerical and experimental curves to fit well, as depicted in Fig. 12b and 14. Figure 14a reports the numerical and experimental evolution in time of the total amount of complex molecules. Compared with Fig. 9a, the plot does not exhibit significant differences neither in terms of complex generation rate nor with regard to the shape of the curves. In fact, both plots are well within the error bars in the experimental data. The initial concentration of immobile receptors has been taken as the $23\%$ of VEGFR2, i.e., $c_{\mathrm{R^i}_R}^{0}=4.393$ $\mathrm molecules/\mu m^2$ and $c_{\mathrm{R^m}_R}^{0}=14.707$ $\mathrm molecules/\mu m^2$.

Nevertheless, the in silico trial that accounts for immobile VEGFR2 promotes a lower amount of complexes at the end of the simulations, even though the quantity of available ligands is unaltered (compare Fig. 13a and 14b). Whereas free diffusion of receptors causes the complete depletion of VEGFR2 on the cell membrane for the unvalidated model (see Fig. 12a), the numerical solution of eqs. (28), owing to the presence of immobile species, shows an amount of receptors at the apical side of the cell that is coherent with the experimental observation (see Fig. 12b), thus complying with the biological request that the residual amount of free receptors in the apical membrane after 7200 s is approximately $30\%$ of the initial concentration.

## Conclusions

Taking advantage of experimental data and numerical simulations, we investigated the recruitment of VEGFR2 during EC adhesion to its specific ligand adsorbed onto ECM. We argued that the electrostatic interactions are not sufficient to induce ECs spreading. This statement was proved experimentally, observing that ECs poorly spread onto Poly-Lysine ECM, which prevents the cytoskeleton reorganization. This evidence unveils that the EC spreading, and eventually the EC motility, shall be attributed to a complex series of cellular processes, such as the membrane protrusion at the leading edge and the adhesion to the extracellular microenvironment, followed by retraction and contraction. A model of cell migration, capable of capturing the micro-structural details of those processes, is currently not available. This paper did not attempt at proposing such a motility framework, either. Rather, we focused on the relocation of proteins along the advecting membrane during migration, since the recruitment of growth factor receptors at the leading edge may drive cells during directional migration in tissues.

In this work, we surrogated the cytoskeletal machinery through the setup and evolution in time of purposely designed bulk forces, oriented axis-symmetrically, replicating the constitutive laws that link cytoskeletal strains to internal stresses. In this way, the mechanical response and the protein relocation are only one-way coupled, i.e., the latter does not influence the cell deformation.

Comparing co-designed experiments and simulations, we achieved a neat *qualitative and quantitative* understanding of the processes that preside the relocation of VEGFR2 on the membrane, and established parameters that could hardly be measured. We eventually tailored the model to reproduce advanced features, as the residual number of free receptors in the apical side of the cell after the mechanical deformation phase completes.

Further developments are currently in progress in order to grasp the realism of the cytoskeletal reorganization within a rigorous, thermodynamically based multi-physics model. The qualitative understanding of the most relevant multiscale mechanisms that allow cell motility has been achieved after experimental investigations in human neutrophils and other model organisms (Svitkina and Borisy 1999; Keren et al. 2008). Models for cytoskeleton reorganization (Deshpande et al. 2008, 2007; Ronan et al. 2014) will be considered in future works. They entail focal adhesion and hence are coupled with the dynamics of integrins, the actin filament reorganization, transient membrane protrusions and retractions. Globular actins (G-actin) self-assemble into filaments (F-actin) forming polymer networks or bundle to form stress fibers. This dynamic behavior presides the generation and evolution of the mechanical force required by cell motility (Bonanno et al. 2023) Those forces enable forward protrusion of the cell as new actin subunits are added to the fronts of anchored filament bundles. These events are greatly influenced by: i) the stiffness of the ECM; ii) the strength of the cohesive adhesion forces; iii) the relocation and recruitment of integrin on the plasma membrane during advection (Serpelloni et al. 2020) and the interplay with growth factor receptors and other proteins in the ECM. In future research, we will focus on modeling the crosstalk between VEGFR2, $\alpha _v\beta _3$ integrin (Ravelli et al. 2015) and eventually other co-receptors, including ve-Cadherin Carmeliet et al. (1999) and neuropilin Peach et al. (2018) in the angiogenic response during EC migration and proliferation.

## Data Availability

All data, codes, and materials are available upon request.

## A Nomenclature

### Geometrical variables

- – ${\vec {e}}_j$ with $j \le n$ denotes the j*th* unit vector of the canonical basis for $\mathbb {R}^n$
- – $\Omega (t)$ and $\Omega _R$ denote the cell domain in current and reference configuration, respectively
- – $\partial \Omega (t)$ and $\partial \Omega _R$ denote the cell membrane domain in current and reference configuration, respectively
- – ${ {{\mathcal {P}}} }$ and ${ {{\mathcal {P}}} }_R$ denote a generic subpart of $\partial \Omega (t)$ and $\partial \Omega _R$, respectively
- – $\vec {n}$ and $\vec {n}_R$ denote the binormal vector that identify the normal direction at a given place belonging to the advecting and referential surface, respectively
- – $\vec {\tau }_{\parallel }$ and $\vec {\tau }_{\bot }$ denote the tangent and the outward normal unit vectors forming together with $\vec {n}$ the basis of the Frenet frame at a given place belonging to the advecting cell surface
- – *s* denotes the arc length measured on $\partial \mathcal{P}(t)$
- – $g_N$ denotes the minimum distance between a point on the cell membrane and the glass-made $\mu$slide

#### Operators

- – The symbol $\textrm{div} \left[ \, {} \, \right]$ and $\mathrm{\nabla } \left[ \, {} \, \right]$ denote the divergence and gradient operators in current configuration
- – The symbols $\textrm{Div} \left[ \, {} \, \right]$ and $\textrm{Grad} \left[ \, {} \, \right]$ denote the same operators in reference configuration
- – The symbols $\textrm{div}_{ {{\mathcal {P}}} } \left[ \, { } \, \right]$ and $\mathrm{\nabla }_{ {{\mathcal {P}}} } \left[ \, { } \, \right]$ denote, in current configuration, the divergence and gradient projected operators on the subpart ${{\mathcal {P}}}$ of the surface $\partial \Omega (t)$ of an advecting cell volume $\Omega (t)$
- – The symbols $\textrm{Div}_{ {{\mathcal {P}}}_R } \left[ \, { } \, \right]$ and $\textrm{Grad}_{ {{\mathcal {P}}}_R } \left[ \, { } \, \right]$ denote the same operators in reference configuration
- – The symbol $\cdot {}$ denotes the scalar product, either for vectors or for tensors ( double index contraction )
- – The symbol $\frac{ \textrm{d} \phi }{\textrm{d} t}$ denotes the *total* derivative of a field $\phi$ (scalar, vector, or tensor) with respect to time *t*, while $\frac{\partial \phi }{ \partial t}$ denotes the *partial* derivative of a field $\phi$ (scalar, vector, or tensor) with respect to time *t*
- – The symbol $| {\cdot } |^2$ denotes the squared norm of a vector $\vec {x}$ or a tensor ${\varvec{x}}$

#### Variables and fields

***Mechanical variables and fields***

- – ${\varvec{F}}$ denotes the gradient strain tensor
- – ${\varvec{P}}$ denotes the first Piola–Kirchhoff stress tensor
- – $\vec { u}$ denotes the displacement vector
- – $\vec { t}$ denotes the surface force vector
- – $\vec { b}$ denotes the body force vector
- – *J* denotes the determinant of the gradient strain tensor
- – $p_N$ denotes the normal component of a generic surface force vector

***Mass transport variables and fields***

- – $c_{\beta }$ and $c_{{\beta }_R}$ denote the concentration measure called molarity (moles per unit area) for the $\beta$-th species in current and reference configuration, respectively
- – $c_{\mathrm{\beta }_R}^{0}$ denotes the initial concentration for the $\beta$-th species
- – ${c_{\mathrm{\beta }}^\mathrm{{max}}}$ and ${c_{\mathrm{\beta }_R}^\mathrm{{max}}}$ denote the saturation limits for the $\beta$-th species in current and reference configuration, respectively
- – $\theta _\beta$ and $\theta _{\mathrm{\beta }_R}$ denote the effective concentration of the $\beta$-th species in current and reference configuration, respectively
- – ${s}_\beta$ and ${s}_{\mathrm{\beta }_R}$ denote the mass supply rate of species $\beta$, the number of moles of species $\beta$ per unit area per unit time in current and reference configuration, respectively
- – $w^{}$ and $w_R^{}$ denote the reaction rate of a specific chemical reaction in current and reference configuration, respectively
- – $\vec {h}_{\beta }$ and ${ { \vec {h}}_{\mathrm{\beta }_R} }$ denote the mass flux of species $\beta$, i.e., the number of moles of species $\beta$ per unit line per unit time in current and reference configuration, respectively

***Other variables and fields***

- – *t* denotes time
- – $T$ denotes the absolute temperature
- – $\alpha$ and $\alpha _R$ denote the kinetic "parameter" in current and reference configuration, respectively

#### Constants and parameters

- – CA denotes the contact area between the cell membrane and the glass-made $\mu$slide
- – $\mathcal {R}$ denotes the universal gas constant
- – ${k_{f}}$ and ${k_{f_R}}$ denote the forward “constants” of a specific chemical reaction in current and reference configuration
- – ${k_{b}}$ and ${k_{b_R}}$ denote the backward “constants” of a specific chemical reaction in current and reference configuration
- – $K_\textrm{eq}^{}$ denotes the equilibrium constant of a specific chemical reaction
- – $\Delta G^{0}$ denotes the standard Gibbs free energy of reaction
- – $\ell _{chem}$ denotes chemical length-scale that tunes the amount of available ligands to the gap $g_N$
- – $c_{{\textrm{L}}_{\textrm{R}}}^{av}$ denotes the maximum amount of available ligands on the cell membrane
- – $\textrm{D} \!\! | \,_R$ denotes the diffusivity of Vascular Endothelial Growth Factor Receptor 2 (VEGFR2)
- – $\kappa$ denotes the bulk modulus
- – *G* denotes the shear modulus
- – $\rho _{rl}$ denotes the minimum concentration of receptors and ligands at a location on the cell membrane

## B Materials and methods

### B.1 The in vitro protocol

**Cell cultures** - Human umbilical vein endothelial cells (HUVECs) were grown in M199 medium (Gibco, Life Technologies, Grand Island, NY) enriched with 20% fetal calf serum (FCS, Gibco, Life Technologies), EC supplement (100 mg/mL) (Sigma Chemical Co., St. Louis, MO) and porcine heparin (Sigma) (50 $\mu$g/mL). HUVECs were used at early passages (I-IV) and grown on plastic surfaces coated with porcine gelatin (Sigma). Fetal bovine aortic endothelial GM7373 cells were grown in Dulbecco’s modified Eagle medium (DMEM, Gibco, Life Technologies) containing 10% FCS, vitamins, essential and non-essential amino acids. GM7373 cells were transfected using polyethylenimine (PEI) with pcDNA3/Enhanced Yellow Fluorescent Protein (EYFP) vector harboring the extracellular domain of murine VEGFR2 (ECD-VEGFR2) cDNA (provided by K. Ballmer-Hofer, PSI, Villigen, Switzerland).

**Cell adhesion assay** - Glass coverslips were coated with gremlin (2 mg/mL), fibrinogen (FG) (2 mg/mL) or Poly-L-Lysine (Poly-L) (10 mg/mL) overnight at 4$^\textrm{o}$C. Rat gremlin was produced as described in Mitola et al. (2022). ECs (75.000/cm$^2$ in medium containing 1% FCS) were allowed to adhere to the coverslips for 2 h. Cells were observed under an inverted photomicroscope and phase-contrast snap photographs were digitally recorded for 2 h. Afterward, cells were fixed with paraformaldehyde 4% (PAF) and the F-actin network was stained with AlexaFluor594-Phalloidin and analyzed using a Zeiss Axiovert 200 M epifluorescence microscope equipped with a Plan-Apochromat 63x/1.4 NA oil objective and ApoTome system. Adhesion area was measured using ImageJ software.

The analysis of the cell adhesion area was made by manually drawing the area of a single cell in contact with the coverslip surface using ImageJ measurement tool (https://imagej.nih.gov/ij/download.html). For fibrinogen (FG) and poly-l-lysine, 40–60 cells were analyzed in each sample. For cells in suspension, images were acquired above the coverslip plane and again the cell area was calculated by drawing the contours of the cells. Samples were analyzed for statistical significance using unpaired two-tailed t test. One asterisk means $P<0.05$, while four asterisks mean $P < 0.0001$. The error bars represent the Standard Error of Mean.

**VEGFR2 relocation assay** - Enhanced yellow fluorescent protein (EYFP)-tagged extracellular domain (ECD) of VEGFR2 (ECD-VEGFR2-EYFP) expressing GM7373 cells were cultured on coverslips. Coverslips were then flipped on gremlin or fibrinogen (FG)-coated microslides and analyzed for 120 min. Z-stack images in time lapse were recorded using a Zeiss Axiovert 200 epifluorescence microscope equipped with a Plan-Apochromat 63$\times$/1.4 NA oil objective and ApoTome system. Images in Fig. 4 show ECD-VEGFR2-EYFP distribution at the basal portion of cells in contact with the FG- or gremlin-coated surface at 30 min, cell orthogonal z reconstructions, and 3D reconstructions. Graph in Fig. 5 represents the quantification of normalized fluorescence of ECD-VEGFR2-EYFP on fibrinogen- (orange line) or gremlin-coated (blue line) surfaces during cell adhesion. Data represent the mean of 6 different cells Ravelli et al. (2015).

## Footnotes

1 The difference in size could be due to different sample preparation techniques, as some authors suggest. EC isolation procedures for culture may deteriorate the fragile glycocalyx, and in vitro ECs are unable to reconstruct the long glycocalyx by themselves in terms of quantity and/or quality of constituents (Chappell et al. 2009; Florian et al. 2003).

2 To express the extracellular domain of VEGFR2 tagged with the fluorescent proteins Enhanced Yellow Fluorescent Protein (EYFP) shown in green in Fig. 4b.
